# Acute Testicular Ischemia following Endovascular Abdominal Aortic Aneurysm Repair Identified in the Emergency Department

**DOI:** 10.1155/2014/591820

**Published:** 2014-07-09

**Authors:** Nathan Finnerty, Stephen Rancour, Andrew King

**Affiliations:** ^1^Wexner Medical Center at The Ohio State University, Columbus, OH 43210, USA; ^2^Clinical Affiliate Faculty, Department of Emergency Medicine, Wake Forest University, Winston-Salem, NC 27103, USA; ^3^Clinical Department of Emergency Medicine, Wexner Medical Center at The Ohio State University, Columbus, OH 43210, USA

## Abstract

Endovascular aneurysm repair (EVAR) is perhaps the most widely utilized surgical procedure for patients with large abdominal aortic aneurysms. This procedure is minimally invasive and reduces inpatient hospitalization requirements. The case involves a 72-year-old male who presented to the emergency department with right testicular ischemia two days following EVAR. Given the minimal inpatient hospitalization associated with this procedure, emergency physicians are likely to encounter associated complications. Ischemic and thromboembolic events following EVAR are extremely rare but require prompt vascular surgery intervention to minimize morbidity and mortality.

## 1. Introduction

Endovascular aneurysm repair (EVAR) is becoming the first-line treatment option for many patients with large abdominal aortic aneurysms (AAA) [1]. Ischemic and thromboembolic events following EVAR are extremely rare but may present an emergent threat to life and limb [2, 3]. Recognition of such complications in the emergency department is crucial as these events are more likely to present out of hospital given the reduced hospitalization requirements following EVAR and the potential need for emergent vascular surgical intervention to reduce morbidity and mortality related to these events [1–3]. This paper reviews the epidemiology, clinical presentation, and management of acute testicular ischemia following EVAR. After thorough review of the literature, this is only the second such report in the United States and the third internationally.

## 2. Case Presentation

A 72-year-old Caucasian male with a history of abdominal aortic aneurysm presented to the emergency department two days after abdominal aortic endovascular stent grafting. He presented with right flank, right lower abdominal, and right testicular pain. He reported persistent pain following surgery and was discharged from the hospital less than 24 hours prior to presentation on a bowel regimen and pain medication. Despite regular bowel movements and pain medication consumption, the pain persisted leading to his emergency department visit. He denied any recent fever, chills, headaches or vision changes, chest pain, shortness of breath, nausea, vomiting, dysuria, hematuria, or diarrhea. The pain is not improved with lying flat nor is the patient sexually active.

On physical examination, his heart rate, blood pressure, respiratory rate, and temperature were within normal limits. Pertinent examination findings included a soft, exquisitely tender right testicle in vertical alignment with normal positioning and intact cremasteric reflex bilaterally. The pain persists despite testicular elevation. The abdomen was mildly distended with diffuse tenderness that was worse on the right. No peritoneal signs or costovertebral angle tenderness were noted. Femoral and dorsalis pedis pulses were present and equal bilaterally.

**Figure 1:**
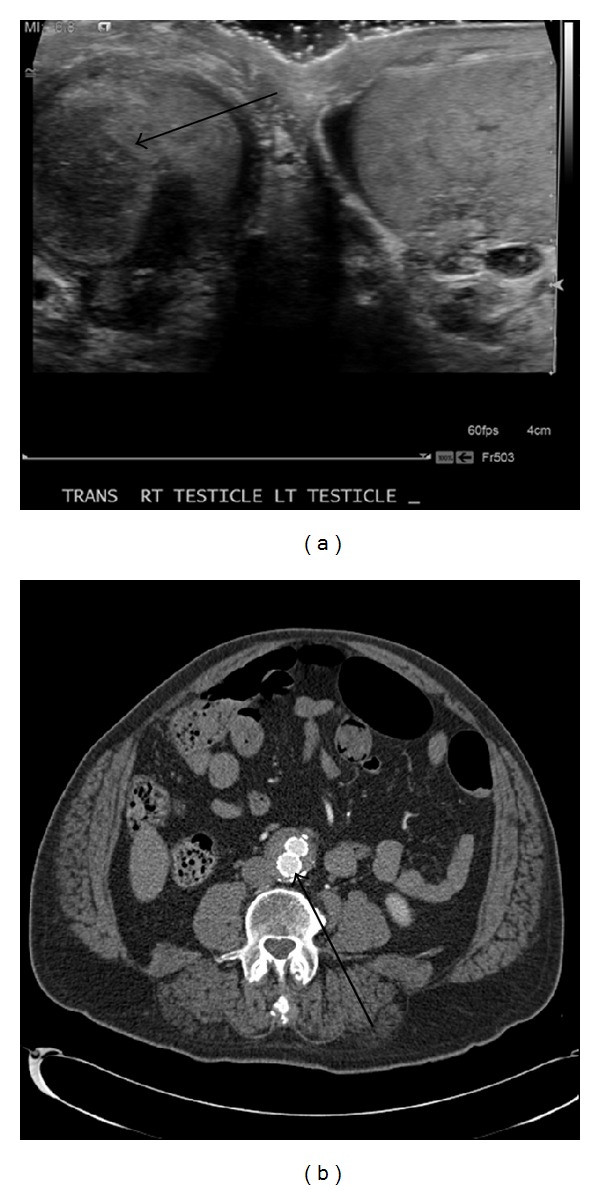
(a) Testicular ultrasound demonstrating decreased echogenicity within the right testicle (arrow). (b) Computed topographic angiogram (CTA) showing endovascular stent graft (arrow).

Urology was consulted with coinciding history and physical exam findings. Following their clinical examination, urology recommended against performing emergent manual detorsion because the presentation was not consistent with testicular torsion. The patient subsequently underwent ultrasonographic evaluation (Figure 1(a)) of the scrotum and testicles, revealing a mildly enlarged right testicle with decreased echogenicity and decreased flow as compared to the left, with concern for infarct given his recent vascular surgery.

Intermittent torsion was considered less likely. This was followed by a computed topographic angiogram (CTA) study (Figure 1(b)), which read: “status post aortobi-iliac endograft. No endoleak identified. Aneurysmal dilatation of the infrarenal aorta, bilateral common iliac arteries, and left common femoral artery, consistent with previous imaging.” On further review, the right limb of the stent graft extended above the hypogastric artery origin, though the artery itself was widely patent. All information and findings being considered, it was concluded that the patient had suffered testicular ischemia as a complication of EVAR from wither a thromboembolism from the hypogastric artery into the testicular artery or reduced blood flow from the graft itself. He was managed conservatively with symptom control and observation with serial examinations. Repeat testicular ultrasound showed stable blood flow. His symptoms improved and he was discharged from the hospital three days following his emergency department presentation.

## 3. Discussion

An abdominal aortic aneurysm (AAA) is an abnormal dilation of the major abdominal artery found in 5% to 10% of men aged 65 to 79 years [4, 5]. Several complications are associated with AAA, the most severe being rupture. The estimated mortality after rupture is 80% for those reaching the hospital and 50% for those undergoing surgery, with combined prehospital and inpatient mortality estimates as high as 95% [2]. Ultrasound screening for men aged 65 to 79 years is recommended with elective surgical intervention for aneurysms greater than 5.5 cm in diameter or for symptomatic aneurisms, given proven mortality benefits [4, 5].

Surgical repair of AAA can be attained via open surgical repair (OSR) or endovascular stent grafting known as endovascular aneurysm repair (EVAR) [1]. EVAR is less invasive when compared to traditional OSR and can be performed with regional rather than general anesthesia. It has demonstrated lower short-term mortality and is associated with reduced operative time, shorter intensive care requirements, and less blood loss than OSR [1]. Average hospital stay following EVAR was found to be 3–6 days [2]. As such, EVAR has gained popularity as the primary intervention for AAA over the past two decades [1].

Of the known complications of EVAR, leakage of blood into the aneurysm sac (endoleak) is most common. Additional complications include stent migration, aneurysm sac expansion, stent occlusion, and delayed rupture [1, 6]. Ischemic complications following EVAR are uncommon but have included acute limb ischemia due to stent graft limb occlusion as well as colon and bladder ischemia [6–10]. Thromboembolic events are also rare but have been reported in association with both EVAR and OSR [2, 3, 6].

In a case report by McKenna et al., a man of similar age presented with acute onset left scrotal pain following EVAR [6]. One notable difference for McKenna et al. was that the patient presented 6 weeks after operation and had undergone CTA surveillance at 4-week follow-up, as opposed to 2 days after procedure reported here. Initial evaluation included testicular ultrasound which revealed absence of blood flow through the left testicular artery to the left testis. The patient subsequently underwent emergent left orchiectomy for infarcted testicle. Hall et al. report a case of left testicular ischemia following EVAR and confirmed by ultrasonography [7]. As in the case presented here, Hall et al. report symptom onset within 7 days postoperatively and conservative management after the patient declined orchiectomy; however, outcomes differed in that they report ischemia with progression to infarction [7].

In the case presented, acute ischemia was suspected clinically and confirmed ultrasonographically. Blood flow to the right testicle was reduced with right testicular decreased echogenicity following EVAR, without clinical signs of torsion or infarction. As opposed to the previously mentioned cases, this patient was managed conservatively with symptom control and close observation and required no surgical intervention. Repeat ultrasound demonstrated stable blood flow to the right testicle and symptoms had resolved prior to discharge.

The exact pathophysiology of testicular ischemia and infarction following EVAR is not clear as this is a rare complication with scarce literature. McKenna et al. [6] report thrombus as the inciting event; Hall et al. [7] consider the absence of adequate collateral blood flow, delayed occlusion of collaterals, and progressive thrombosis of the aneurismal sac. In reviewing current literature, we add that graft migration and thromboembolism are also potential explanations of the case presented [2, 3, 8–10]. The variation in postoperative onset of symptoms is explained by the number of potential causes and further reports and studies are needed to pinpoint the exact or most common mechanism. This report is targeted to the emergency physician with the objective to increase awareness, broaden differential diagnostics, and optimize patient care as ischemic complications following EVAR are most likely to present in the emergency department.

## 4. Conclusion

Endovascular aneurysm repair is becoming the primary treatment option for many patients with operative AAA. Given the decreased intraoperative complications and reduced inpatient requirements, postoperative complications are more likely to occur at home and present to the emergency department for evaluation. These patients should be evaluated closely and in conjunction with vascular surgeons for potential end-organ damage secondary to acute ischemia and thromboembolic events.

## References

[1] Paravastu SC, Jayarajasingam R, Cottam R, Palfreyman SJ, Michaels JA, Thomas SM (2014). Endovascular repair of abdominal aortic aneurysm. *Cochrane Database of Systematic Reviews*.

[2] Prinssen M, Verhoeven ELG, Buth J (2004). A randomized trial comparing conventional and endovascular repair of abdominal aortic aneurysms. *The New England Journal of Medicine*.

[3] EVAR trial participants (2005). Endovascular aneurysm repair versus open repair in patients with abdominal aortic aneurym (EVAR trial 1): randomised controlled trial. *The Lancet*.

[4] Filardo G, Powell JT, Martinez MA, Ballard DJ (2012). Surgery for small asymptomatic abdominal aortic aneurysms. *Cochrane Database of Systematic Reviews*.

[5] Cosford PA, Leng GC (2007). Screening for abdominal aortic aneurysm. *Cochrane Database of Systematic Reviews*.

[6] McKenna AJ, Gambardella I, Collins A, Harkin DW (2009). Testicular infarction: a rare complication of endovascular aneurysm repair treatment for aortoiliac aneurysm. *Journal of Vascular Surgery*.

[7] Hall MJ, Duprat GI, Poulin TL (2010). Testicular ischemia following endovascular infrarenal abdominal aortic aneurysm repair: a rare complication. *Journal of Vascular and Interventional Radiology*.

[8] Zander T, Baldi S, Rabellino M (2007). Bilateral hypogastric artery occlusion in endovascular repair of abdominal aortic aneurysms and its clinical significance. *Journal of Vascular and Interventional Radiology*.

[9] Rayt HS, Bown MJ, Lambert KV (2008). Buttock claudication and erectile dysfunction after internal iliac artery embolization in patients prior to endovascular aortic aneurysm repair. *CardioVascular and Interventional Radiology*.

[10] Perry RJT, Martin MJ, Eckert MJ, Sohn VY, Steele SR (2008). Colonic ischemia complicating open vs endovascular abdominal aortic aneurysm repair. *Journal of Vascular Surgery*.

